# The Influence of Recombinant Bovine Somatotropin (rbST) on the Metabolic Profile and Milk Composition of Lactating Murrah Buffalo

**DOI:** 10.3390/ani14040636

**Published:** 2024-02-16

**Authors:** Marcelo Arne Feckinghaus, Mariana Guimarães de Oliveira Diogo, Vanessa Martins Storillo, Fabio Celidonio Pogliani, Bruno Moura Monteiro, Paulo Fantinato Neto, Melina Marie Yasuoka, Daniela Becker Birgel, Eduardo Harry Birgel Junior

**Affiliations:** 1 Department of Internal Medicine, School of Veterinary Medicine and Animal Sciences, University of São Paulo, 87 Professor Orlando Marques de Paiva Avenue, São Paulo 05508-010, SP, Brazil; fabiocp@usp.br (F.C.P.); brunomouramonteiro@hotmail.com (B.M.M.); 2 Department of Surgery, School of Veterinary Medicine and Animal Sciences, University of São Paulo, 87 Professor Orlando Marques de Paiva Avenue, São Paulo 05508-010, SP, Brazilveterinariavanessa@yahoo.com.br (V.M.S.); fantinatont@gmail.com (P.F.N.); melinamarie@gmail.com (M.M.Y.); 3 Department of Veterinary Medicine, Faculty of Animal Science and Food Engineering, University of São Paulo, 225 Duque de Caxias, Pirassununga 13635-900, SP, Brazil; dabirgel@usp.br

**Keywords:** bovine somatotropic hormone, rbST, metabolic parameters, milk yield

## Abstract

**Simple Summary:**

This study was undertaken to evaluate the effects of a single subcutaneous dose of 500 mg rbST on the lipid profile, liver and kidney function, and physical, chemical, and cellular constitution of buffalo milk. Our data indicated that the use of rbST in buffalo from the 100th day of lactation is metabolically safe since the treatment neither caused imbalances in fat metabolism nor overloaded the liver or renal function. The changes in milk composition were transient and limited to a decrease in milk protein.

**Abstract:**

The use of recombinant bovine somatotropin (rbST) leads to an increase in variable amounts of milk production in buffalo, but there is a lack of information on the influence of rbST on their metabolism. This study looked at the effects of a single 500 mg dose of rbST on the lipid profile, liver and kidney function, and physical, chemical, and cellular constitution of milk in 14 buffalo over 14 days, from the 100th day of lactation, compared with 14 animals in a control group. From the first day after rbST, there was a rise in beta-hydroxybutyrate (β-HBO), possibly due to higher dry matter intake or the biotransformation of NEFA into β-HBO. The treatment did not influence blood glucose, non-esterified fatty acids (NEFAs), triglycerides, cholesterol, total protein, albumin, AST, GGT, bilirubin, urea, or creatinine levels. In 71.3% of the buffalo, there was a gradual increase in milk production, with the maximal response occurring in the first week followed by a gradual decrease, whilst in 21.4%, the increase in production occurred between 7 and 10 days. Only 7.1% of the animals did not respond. On the 3rd, 5th, 7th, and 10th days after treatment, an increase was found in daily milk production between the two groups equal to 1.04, 1.52, 1.42, and 1.06 L, respectively. In relative terms, this means an increase in milk production, respectively, of 15.1%, 21.0%, 19.8%, and 15.1%. The constitution of the milk showed no difference in the amounts of fat, lactose, total solids, or somatic cell count; however, on the third day after rbST administration, there was a decrease in protein. Notably, from the fifth day, the protein values showed no statistical difference. It can be concluded that the use of rbST in buffalo from the 100th day of lactation is metabolically safe since the treatment neither caused imbalances in fat metabolism nor overloaded the liver or renal function, and the changes in milk composition were transient and limited to a decrease in milk protein.

## 1. Introduction

Buffalo milk has a high nutritional content and yield for the production of its derivatives, and, in recent decades, there has been an increase in the consumption of dairy products from buffaloes, leading to an increase in appreciation of the buffalo species in dairy farming worldwide [1,2]. Given this fact, producers are looking for techniques to increase milk production and the proportion of total solids for industrialization [3]. The administration of recombinant bovine somatotropin (rbST) is a technology that has been important for dairy cattle breeders, as it directly impacts the profitability of the system [4].

The use of rbST in female cattle leads to gradual increases in milk production a few days after administration, with the maximum response reached within the first week [5]. In addition, there is an increase in lactation persistence, preventing a sharp drop in production after peak [6]. After discontinuation of rbST treatment, there is a gradual decrease in milk production, eventually returning to the levels recorded before the beginning of its use; however, repeating the treatment every 14 days causes the increase in milk production observed in the first week to be sustained [5].

Recombinant bovine somatotropin is biologically active in sheep [7], goats [8], and buffalo [9]. In buffalo, there has been a significant variation in the dosage and interval of repeated use of this hormone, showing an overall increase in milk production of between 9.8 and 62.6% at doses ranging between 250 and 640 mg of rbST, repeated every 14, 21, or 28 days [7,9,10,11,12,13,14,15,16].

In studies assessing the influence of rbST in milk composition, alterations in fat and protein were not found when the animals were in a positive energy balance [9,10,11,17], while the somatic cell count showed enormous variation, which does not allow any statement about the effects of the treatment [10].

The shortage of information about the influence of rbST on the metabolism of buffalo motivated the development of this research in order to evaluate the effects of a single subcutaneous dose of 500 mg of rbST on the lipid profile, liver and kidney function, and physical, chemical, and cellular constitution of buffalo milk.

## 2. Materials and Methods

### 2.1. Ethical Statement

All aspects of this study were performed with the approval of the Bioethics Committee of the School of Veterinary Medicine and Animal Sciences, University of São Paulo, São Paulo, Brazil (Protocol No. 1405/2008).

### 2.2. Animals

A total of 28 healthy female Murrah (Bubalus Bubalis) buffaloes that were in good body condition (BCS varying between 3.00 and 3.50), weighing 575.5 ± 12.5 kg, without mammary gland diseases, and with eutocic deliveries and that were between 100 and 200 days of lactation (141.6 ± 4.2 days in milking), were used in this study. During the experimental period, the animals were kept in a semi-intensive breeding system in Brachiaria ruziziensis pastures. Panicum maximum silage, composed of cottonseed (2 kg/day), brewer residue (10 kg/day), and citrus pulp (2 kg/day), was used as roughage. The average composition of the concentrate was 20.8% crude protein and 73% total digestible nutrients (TDN), with an estimated average consumption of 190 g of crude protein and 660 g of TDN per liter of milk produced.

In this prospective observational study, two groups of female buffalos were assigned to groups according to the type of treatment. One group consisted of female buffalos (*n* = 14) that received recombinant bovine somatotropin (Boostin^®^, Schering-Plough Animal Health, Sao Paulo, Brazil, 500 mg, subcutaneously, in ischiorectal fossa), and a second group (*n* = 14) consisted of female buffalos that did not receive treatment of any kind (control group).

The animals were machine-milked twice a day with an average interval of 12 h between milking. The animals had been averaging a total lactation of 2000 to 3000 kg in previous lactations. Milk production was recorded on the day of sampling using the MK-V Waikato Milk Meter (Waikato Milking Systems, Horotiu, New Zealand).

### 2.3. Sampling

The animals were examined and sampled (blood and milk) at the following times: immediately before the use of rbST and on the 1st, 3rd, 5th, 7th, and 14th days after the use of rbST.

Blood samples were taken by puncture of the external jugular vein into a tube without anticoagulant (Becton Dickinson Vacutauner^®^ Systems, Frankin Lakes, NJ, USA). The samples were placed in ice and sent to the laboratory within 2 h of collection and were centrifuged at 1000× *g* for 150 min for serum separation. The supernatant serum was stored at −20 °C until analysis.

The total serum protein was determined using the biuret method. The albumin was tested via the bromocresol green method. The aspartate transaminase (AST), gamma-glutamyl transpeptidase (GGT), cholesterol, and triglycerides were determined using a commercial kit, BioSystems Reagents & Instruments, Barcelona, Spain. Bilirubin levels were determined using a commercial kit, Labtest, Minas Gerais, Brazil. Non-esterified fatty acids (NEFAs) and beta-hydroxybutyrate (β-HBO) were quantified with a commercial kit, Randox Laboratories, Dublin, Ireland. Blood glucose and urea were determined with a commercial kit, Diasys Diagnostic Systems, Holzheim, Germany. Serum creatinine levels were measured using a commercial kit, Labtest, Minas Gerais, Brazil. All biochemical determinations were quantified at 25 °C in a Biochemical Analyzer Liasys model, AMS, Caserta, Italy.

For the analysis of the physical, chemical, and cellular constitution of the buffalo milk, 40 mL of milk was collected in plastic vials containing preservative tablets of bronopol (2-bromo-2-nitropropane–1.3-diol). Before the start of the somatic cell count and determination of the amounts of lactose, fat, protein, and total solids, the milk samples were kept in a water bath at 38 °C for 15 min, then homogenized manually for the determination of lactose, fat, protein, and total solids by infrared radiation using Bentley 2000 equipment (Bentley Instruments Inc., Chaska, MN, USA). The number of somatic cells in the milk was measured by flow cytometry using Somacount 500 equipment (Bentley Instruments Inc.).

### 2.4. Statistical Methods

IBM SPSS Statistics software, version 15.0 for Windows, was used for the statistical analysis. Data were expressed as mean with standard error. Firstly, the Levene test was used to test the homoscedasticity of the variables. After confirming the homogeneity of variances between groups, the analysis of variance (ANOVA) with repeated measures was used. In the repeat measures ANOVA procedure, the group, time, and interaction between group and time factors were tested. The significance level was 5%. If there were statistical differences in the time factor, mean values were compared using the Fisher’s least significant difference (LSD) test. For multiple comparisons, values of *p* less than 0.24% were considered (the result of dividing by 5% by 21 comparisons between times 2 and 2). If there were statistical differences in the group factor, respective intervals with 95% confidence were obtained. When the interaction between group and time was significant, the groups were compared separately at each time by Student’s *t* test.

## 3. Results

**Figure 1:**
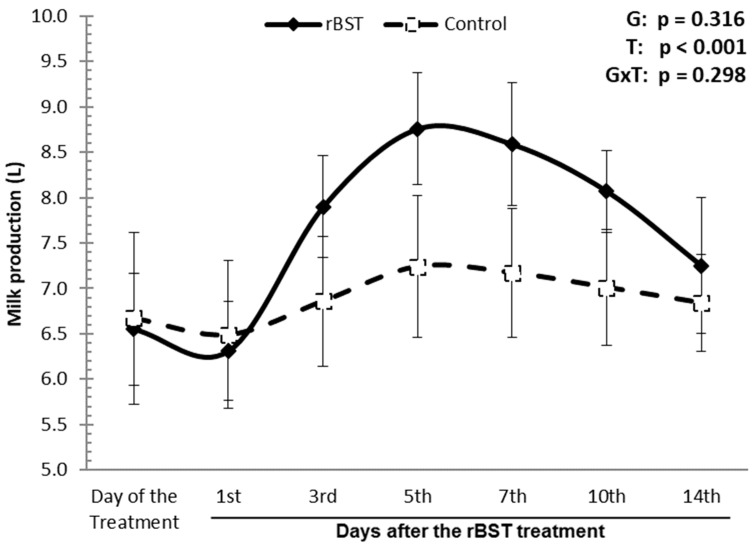
Influence of the use of recombinant bovine somatotropin (rbST) on the amount of milk (L) produced from Murrah buffaloes.

The application of statistical tests could not prove the existence of significant statistical differences between the rBST and control groups (*p* = 0.316); however, when the statistical analysis was performed as a function of the time elapsed after the use of rBST, significant statistical differences were observed (*p* < 0.001). It was found in this analysis that only in the group treated with rBST was there an increase in milk production, while in the control group, there were no variations in milk production in the studied period (Figure 1). It was observed that daily milk production in the group treated with rBST was higher at the following times: day of treatment X 5th day after treatment (*p* = 0.0019); day of treatment X 7th day after treatment (*p* = 0.0020); 1st day after treatment X 5th day after treatment (*p* = 0.0009); and the 1st day after treatment X 10th day after treatment (*p* = 0.0020). In 71.3% of the buffalo treated with rBST, there was a gradual increase in milk production, with the maximal response in the first week, followed by a gradual decrease, whilst in 21.4%, the increase in production occurred between 7 and 10 days, and only 7.1% of animals did not respond. On the 3rd, 5th, 7th, and 10th days after treatment, an increase was found in daily milk production between the two groups equal to 1.04, 1.52, 1.42, and 1.06 L, respectively. In relative terms, this means an increase in milk production, respectively, of 15.1%, 21.0%, 19.8%, and 15.1%.

**Figure 2:**
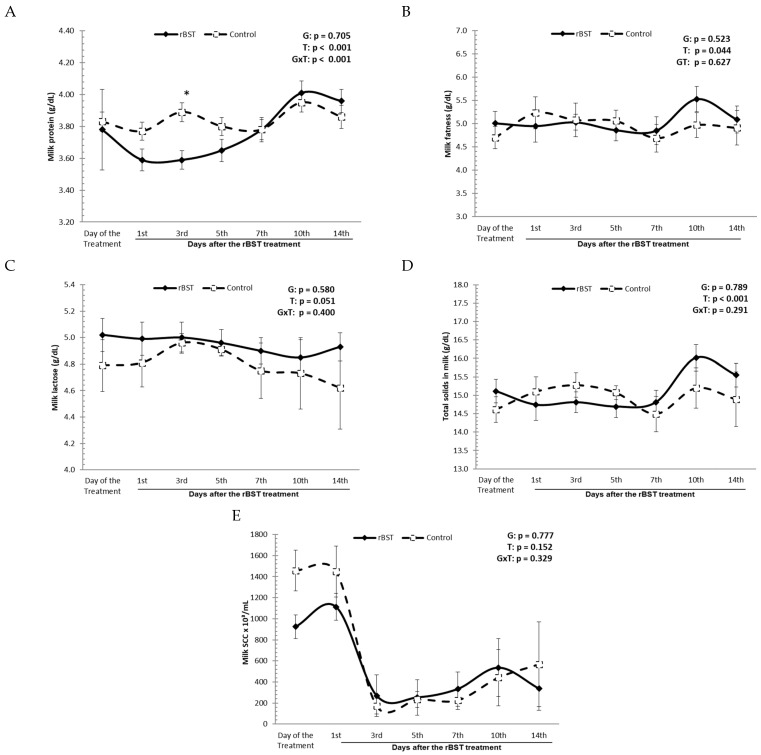
Influence of the use of recombinant bovine somatotropin (rbST) on the physical, chemical, and cellular constitution of Murrah buffalo milk: (**A**) protein milk level; (**B**) fatness milk level; (**C**) lactose milk level; (**D**) total solids milk level; and (**E**) number of somatic cells in the milk. Milk: CCS. * presence of signal denotes the presence of statistical differences between groups.

In the early days after the use of rbST, a decrease in milk protein content was observed, and on the third day, it was found that the milk protein values in the treated group (3.59 ± 0.06 g/dL) were significantly lower than those observed in the control group (3.89 ± 0.06 g/dL). A statistically significant difference was found between the groups on the third day (*p* < 0.0001). From the fifth day after the start of hormone treatment, protein values began to oscillate without any statistical difference between the treated and control groups (Figure 2A).

The existence of a relationship between the influence of the use of rbST on fat, lactose, and total solids content in buffalo milk was not found during the 14 days of the experiment. The fat values, lactose, and total solids content in the milk of the group treated with 500 mg of rbST ranged from 4.85 ± 0.30 to 5.53 ± 0.27 g/dL, 4.85 ± 0.13 to 5.02 ± 0.13 g/dL, and 14.69 ± 0.29 to 16.02 ± 0.36 g/dL, respectively, while in the control group, the fat, lactose, and total solids ranged between 4.69 ± 0.30 and 5.23 ± 0.34 g/dL, 4.62 ± 0.31 and 4.96 ± 0.07 g/dL, and 14.49 ± 0.48 and 15.28 ± 0.33 g/dL, respectively, without any statistical difference observed during the experimental study (Figure 2B–D).

From the analysis of somatic cell counts in the milk of the buffaloes, we verified that there was no influence of the administration of rbST on the number of somatic cells in the samples collected, and there was no statistical difference in the comparison of the experimental groups. During the 14 days of the experiment, the number of somatic cells in the milk in the rbST-treated group ranged between 253.1 ± 166.9 and 1113.3 ± 126.6 × 10^3^; cells/mL, while in the control group, it ranged between 171.8 ± 75.1 and 1457.1 ± 193.7 × 10^3^ cells/mL (Figure 2E).

**Figure 3:**
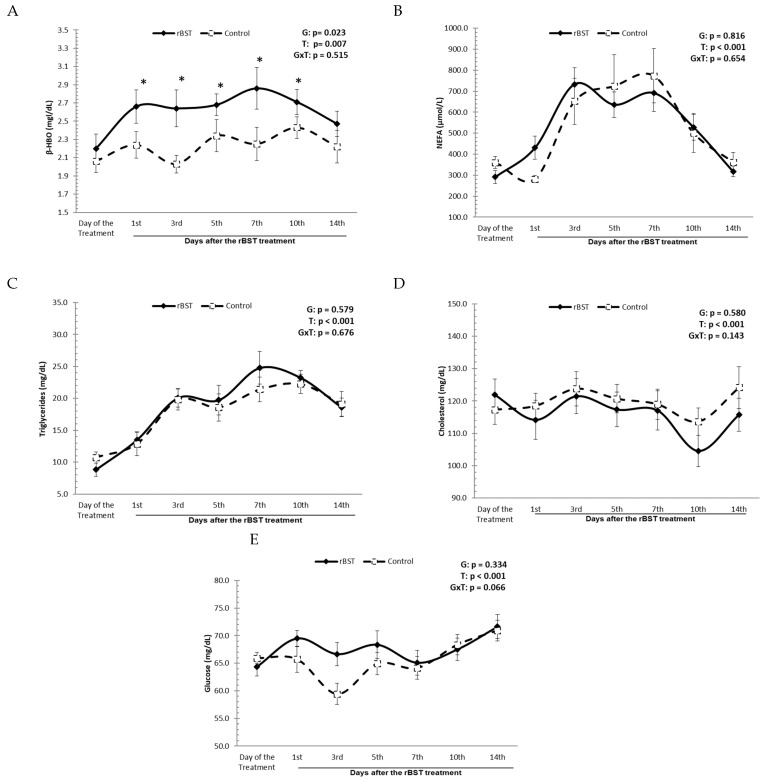
Influence of the use of recombinant bovine somatotropin (rbST) on the lipidogram and plasmatic glucose concentrations of Murrah buffaloes: (**A**) β-HBO serum levels; (**B**) NEFA serum levels; (**C**) triglyceride serum levels; (**D**) cholesterol serum levels; (**E**) plasmatic glucose concentrations. * presence of signal denotes the presence of statistical differences between groups.

Hormonal treatment with rbST had an effect on the lipidogram of buffaloes in relation to the level of ketone bodies in the blood circulation. A statistically significant difference was observed for β-HBO serum levels between the group treated with 500 mg of rbST and the control group (*p* = 0.0007). From the 1st day until the 10th day after the hormonal administration, the β-HBO serum levels in the treated group were higher (2.64–2.86 mmol/L) than those in the control group (2.03–2.43 mmol/L). During this period, the serum levels of β-HBO in the rBST-treated group were between 0.28 and 0.61 mg/dL higher than those found in the control group (Figure 3A).

There were no statistically significant changes in NEFA, triglyceride, and cholesterol levels due to the hormonal treatment. During the experiment, the serum levels of NEFAs in the treated group ranged between 291.6 ± 30.8 and 690.8 ± 87.1 µmol/L, while in the control group, they ranged from 281.1 ± 17.7 to 773.7 ± 129.1 µmol/L. The triglyceride serum levels in the group treated with 500 mg of rbST ranged from 8.8 ± 1.0 to 24.8 ± 2.5 mg/dL, while in the control group with no treatment, the values oscillated from 10.7 ± 0.8 to 22.3 ± 1.5 mg/dL. The cholesterol serum levels in the group treated with 500 mg of rbST ranged from 104.5 ± 4.9 to 121.9 ± 4.9 mg/dL, while in the control group, the values oscillated from 113.6 ± 4.2 to 124.2 ± 6.4 mg/dL. No influence on plasma blood glucose was observed. The plasmatic glucose concentrations in the group treated with 500 mg of rbST ranged from 64.4 ± 1.6 to 71.6 ± 2.2 mg/dL, while in the control group, the values oscillated from 59.4 ± 1.9 to 70.9 ± 1.9 mg/dL.

**Figure 4:**
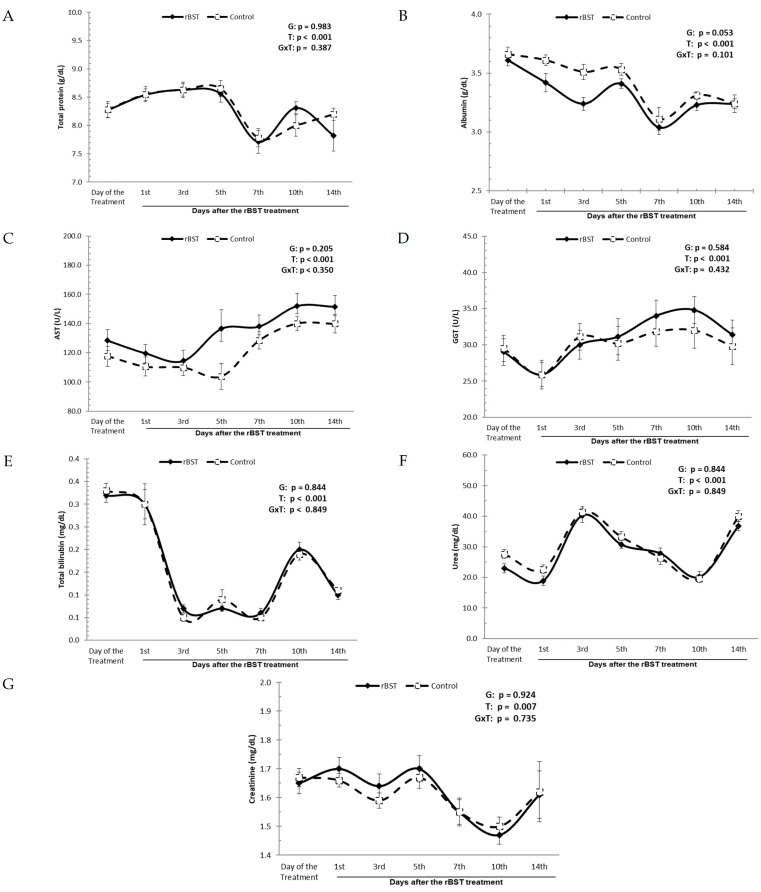
Influence of the use of recombinant bovine somatotropin (rbST) on the liver and renal function of Murrah buffaloes. (**A**) Total protein serum levels; (**B**) albumin serum levels; (**C**) AST serum levels; (**D**) GGT serum levels; (**E**) total bilirubin serum levels; (**F**) urea serum levels; (**G**) creatinine serum levels.

Treatment with rbST had no effect on the liver and renal function of the buffaloes (Figure 4). There were no statistically significant changes in the total protein, albumin, AST, GGT, total bilirubin, urea, or creatinine serum levels due to the hormonal treatment. During the experiment, the serum levels of total protein in the treated group ranged between 7.71 ± 0.20 and 8.63 ± 0.14 g/dL, while in the control group, they ranged from 7.78 ± 0.16 to 8.65 ± 0.14 g/dL. The albumin serum levels in the group treated with 500 mg of rbST ranged from 3.04 ± 0.06 to 3.61 ± 0.05 g/dL, while in the control group with no treatment, the values ranged from 3.11 ± 0.10 to 3.66 ± 0.06 g/dL.

The AST serum levels in the group treated with 500 mg of rbST ranged from 114.4 ± 7.3 to 152.0 ± 8.7 U/L, while in the control group, the values ranged from 103.8 ± 8.8 to 140.0 ± 5.0 U/L. The GGT serum levels in the group treated with 500 mg of rbST ranged from 25.9 ± 1.6 to 34.8 ± 1.9 U/L, while in the control group, the values ranged from 25.9 ± 2.0 to 32.0 ± 2.5 U/L. The serum levels of total bilirubin in the treated group ranged between 0.07 ± 0.01 and 0.32 ± 0.02 mg/dL, while in the control group, they ranged from 0.05 ± 0.01 to 0.33 ± 0.02 mg/dL. The serum levels of urea in the treated group ranged between 18.9 ± 1.5 and 40.3 ± 2.4 mg/dL, while in the control group, they ranged from 19.6 ± 1.3 to 41.3 ± 1.9 mg/dL. The serum levels of creatinine in the treated group ranged between 1.47 ± 0.03 and 1.70 ± 0.05 mg/dL, while in the control group, they ranged from 1.50 ± 0.03 to 1.67 ± 0.04 mg/dL.

## 4. Discussion

In this study, we observed that more than half of the buffaloes showed increased milk production in the first week (between the 3rd and 5th days) after the use of rbST, while a few of the animals had increased milk production in the second week (between the 7th and 10th days) after the hormonal administration. The standard response to the use of rbST found in buffalo in this study was similar to that reported for other bovine animals: gradual increases in milk production a few days after administration, reaching a maximum response during the first week, followed by a gradual decrease in milk production [18]. The percentual increase in milk production after the hormonal administration varied between 15.1% and 21.0%. It was observed that the administration of rbST promoted increases in production that ranged from 3 to 40% [19]. The impact of the use of rBST on increasing milk production in buffaloes was quite variable, as observed in the literature; some authors observed significant increases in milk production [11,15,20], while others did not observe an increase in milk production [17].

This impact on milk production is not directly related to the dose of hormone used since there is research that shows an increase in production with low doses of 250 mg of rBST [15] and research that does not show an increase in production with high doses of 500 mg of rBST [17]. Responses to bST are highly dependent on overall management practices before and after initiation of treatment [7], particularly with the energy density of the diet [16]. Studies report [16] the effect of dietary energy on milk production under the influence of rBST since the daily milk production of high energy density (115% of NCR) is greater than the productions observed at medium energy density (100% of NCR) or low energy density (80% of NCR).

In absolute terms, for the results obtained in our research, the increase in milk production varied between 1.0 and 1.5 L per day in 93% of the buffaloes. This increase was mainly observed between 3 and 10 days after administration (lasting 8 days). Considering that the price of buffalo milk in Brazil for the producer is BRL 3.80 (USD 0.78) and the use of a dose of 500 mg rBST in Brazil is BRL 38.00 (USD 7.80), we could make the following calculation: 8 days versus 1.0 L versus 93% responsive buffaloes = 11.2 L more milk produced, corresponding to a gain of BRL 28.12 (USD 5.80) or 8 days versus 1.5 L versus 93% responsive buffaloes = 7.4 L more milk produced, corresponding to a gain of BRL 42.56 (USD 9.40). Due to the costs of the hormone and the increase in dry matter intake after the use of rBST, its use is not economically viable for buffaloes that produce between 6 and 8 L of milk per day.

The use of rbST makes it possible to direct nutrients to the mammary gland through the action of IGF-I, providing an increase in daily milk production and also an increase in total lactation production [21]. It was verified that there was a significant increase in the amount of milk produced in animals that received rbST, regardless of the use of this hormone for short or long periods [7,22].

During the evaluation of the lipidogram, it was found that between the 1st and 14th days after the use of rbST, the β-HBO serum levels in the treated group were between 0.21 and 0.55 mg/dL higher than those in the control group. Supplementation with rbST for lactating cows led to increased demand for nutrients and dry matter intake [23,24]. Thus, the increase in β-HBO levels observed in this study could be a result of increased food ketogenesis due to an increase in dry matter intake and rumen fermentation processes. Another explanation for the increased values of β-HBO is that they could be the result of the biotransformation of NEFA into β-HBO by an alternative pathway due to a lack of oxaloacetate.

The effects of rbST on lipid metabolism in buffalo reflect the nutritional and/or physiological conditions of the animals undergoing treatment. In the present study, the results revealed that the lipid metabolism of buffaloes treated with rbST remained unchanged, demonstrating that adaptations of fat metabolism, such as adipose tissue mobilization, oxidation and re-esterification of NEFA in the liver, synthesis of triglycerides, and low-density lipoprotein secretion, occurred without overloading lipid metabolism. The lipid metabolism of dairy cows treated with rbST in periods of positive energy balance also did not influence the serum levels of non-esterified fatty acids, triglycerides, and cholesterol [25].

Some authors have reported the occurrence of mild and transient increases in glucose levels in cattle supplemented with rbST, possibly due to increased milk production, with a consequent rise in glucose requirements by the mammary gland for lactose synthesis [21,26,27,28]. Conversely, other researchers have found that the use of rbST does not influence glucose levels in bovine animals [23,29,30]. In this research, buffalo supplemented with rbST had higher plasmatic glucose concentrations on the 3rd day after treatment; however, these differences were not statistically significant, indicating that metabolic homeostasis was maintained. Thus, it appears that, despite the increase in milk production, with a consequent rise in glucose uptake for lactose synthesis in the mammary gland and increased metabolism, animals treated with rbST maintained adequate hepatic gluconeogenesis in order to not interfere with their glycemia.

Adipose tissue also has an important role in glucose concentration since an increase in the mobilization of adipose tissue leads to an increased availability of glycerol and, consequently, an increased availability of glucogenic precursors [26]. rbST acts directly on fat metabolism of the adipose tissue by altering rates of lipolysis and lipogenesis [21,31,32]. The use of 500 mg of rbST on the metabolism of pasture-raised Murrah buffaloes between 63 and 154 days of milking did not change the levels of other energy metabolites (cholesterol and triglycerides) and prosthetics (total protein, urea albumin, and creatinine) [33].

Reports in the literature have shown the influence of rbST on protein concentration, with the treated animals having increased total protein [34] and an associated decrease in albumin [27,35], accompanied by increased levels of globulins [35]. There were no indications from the data obtained in this trial that the hepatic function may have been harmed by treatment with rbST. It was observed that total protein, albumin, AST, GGT, and bilirubin were not influenced by the rbST treatment.

Urea levels are affected not only by physiological factors, such as diet or state of protein metabolism, but also by changes in renal function [36]. Thus, it is essential to combine the values of urea with creatinine, which provides an index of glomerular filtration because of the constant excretion and the fact that they are not strongly affected by the nitrogenous compound content in the diet [37]. An energy deficit could have an effect on metabolism, leading to a decrease in the deamination of amino acids and, consequently, a reduction in serum urea [38,39]. This study did not confirm this possibility because no changes were observed in serum urea levels.

Regarding the constitution of milk from cows supplemented with rbST, most papers did not find changes in their physico-chemical characteristics [21,40,41], and the industrial yield of milk was similar to that obtained from untreated animals [40,42]. In this study, fat values were below that reported in the literature, but they were not affected by rbST treatment, corroborating the papers on buffaloes that did not observe changes in fat content during treatment with rbST [9,10,11,17]. An explanation for the lack of a direct connection between the administration of rbST and the levels of milk fat was the energy status of the animal, since animals in positive energy balance may show no change in the percentage of milk fat different compared to animals in negative energy balance, which may show an increase in the concentration of milk fat. One of the precursors of this constituent of milk (long-chain fatty acids) comes from the lipids circulating in the blood, derived from the diet or from adipose tissue mobilized by rbST [43].

In this paper, the influence of rbST on milk protein content was demonstrated. In the early days after the use of rbST, a decrease in milk protein content was observed, and on the 3rd day, it was found that the milk protein values in the treated group were significantly lower than those in the control group. From the 5th day after the start of the hormone treatment, protein values began to oscillate without any statistical difference between the treated and control groups. This result was in line with the results obtained by Bauman [7], who stated that this decline in milk protein rates is due to the time of the greatest gain and response to milk production in cows supplemented with rbST.

Comparing this study with data from the literature in relation to the concentration of lactose and total solids in milk, the results were in accordance with those reported by several authors [7,17,44], who also did not find any significant difference for these parameters of milk.

For the somatic cell count, a final analysis related to the constitution of the milk was in accordance with that found in cattle [44,45,46] and in buffaloes [17] since the values for this parameter were not influenced by the rbST treatment.

## 5. Conclusions

It can be concluded that the use of rbST in buffaloes from the 100th day of lactation is safe, from a metabolic point of view, since the treatment neither caused imbalances in fat metabolism nor overloaded liver or renal function, and the changes in milk composition were transient and limited to a decrease in milk protein. On the other hand, due to the costs of the hormone and the increase in dry matter intake after the use of rBST, it is considered that its use is not economically viable for buffaloes that produce between 6 and 8 L of milk per day.

## Data Availability

The corresponding author can provide the data that support the findings of this study upon request. The data are publicly available on the following website: https://www.teses.usp.br/teses/disponiveis/10/10136/tde-17022009-091716/publico/Marcelo_Arne_Feckinghaus.pdf (accessed on 14 January 2024).
